# Delayed Presentation of Metachronous Ascending Colonic Adenocarcinoma in Early-Onset Colorectal Cancer Undergoing Curative Resection

**DOI:** 10.14740/jmc5184

**Published:** 2025-12-24

**Authors:** Budhi Ida Bagus, Febriagi Bayu Aji, Nafa Unnisa, Arga Scorpianus

**Affiliations:** a Department of Surgery, Sebelas Maret University, Surakarta, Indonesia; b Medical Faculty, Sebelas Maret University, Surakarta, Indonesia; c Department of Surgery, Moewardi General Hospital, Surakarta, Indonesia

**Keywords:** Early-onset colorectal cancer, Metachronous, Delayed, Resection

## Abstract

Colorectal cancer (CRC) is the third most common cancer in both age groups in the world and one of the increasing causes of mortality each year. Particularly in early-onset colorectal cancer (EOCRC), this age group tends to metastasize to distant organs like the liver, lung, bone, and brain and in metachronous cases. Metachronous cancer in the colorectal adenocarcinoma is usually found in the first 2 - 3 years following curative treatment, with delayed recurrences being extremely rare. We report a rare case of a 42-year-old male with a history of ascending colon adenocarcinoma diagnosed at the age of 31. He underwent curative R0 resection with lymph node involvement, followed by 6 months of adjuvant FOLFOX chemotherapy. He remained disease-free for 11 years under routine surveillance. In 2025, he presented with symptoms of bloody diarrhea and altered bowel habits. Imaging and colonoscopic biopsy revealed a new adenocarcinoma in the distal rectum, representing a delayed metachronous. Clinical staging confirmed localized rectal cancer with limited metastasis (T3N2bM1a). Despite surgical resection being indicated, the patient declined surgery and opted for systemic chemotherapy. He tolerated treatment well, maintained functional status, and reported a good quality of life. Delayed metachronous recurrence can occur in EOCRC even after standard adjuvant therapy and prolonged remission. EOCRC also has a chance to be a delayed metachronous case. Closed follow-up should be performed, particularly in the case of those who refused the operative treatment.

## Introduction

Colorectal cancer (CRC) is the third most frequently diagnosed malignancy and remains a major contributor to global cancer related mortality [1]. Of growing concern is the rising incidence of early-onset colorectal cancer (EOCRC), defined as CRC diagnosed before the age of 50. This subset of patients often presents with advanced stage disease, demonstrates aggressive histopathological characteristics, and is frequently linked to genetic syndromes such as Lynch syndrome or microsatellite instability high (MSI H) status [2, 3]. Distant metastases to the liver, lungs, bones, and brain are not uncommon, and recurrence in EOCRC is often metachronous.

Metachronous CRC refers to the development of a new primary malignancy in the colon or rectum after initial curative intent treatment. It typically occurs within the first 2 - 5 years following surgery [4]. Although a substantial proportion of recurrences occur in this period, delayed recurrence beyond 5 years is rare, especially among younger patients. These atypical cases highlight the limitations of current surveillance protocols and support the need for long-term personalized follow-up [5, 6].

This report presents a rare case of EOCRC with delayed metachronous recurrence 11 years after the initial curative resection and adjuvant chemotherapy, all managed in accordance with National Comprehensive Cancer Network (NCCN) guidelines. This case underscores a unique clinical trajectory and raises important questions about long-term surveillance, treatment decision making, and the biological behavior of EOCRC in younger adults.

## Case Report

A 42-year-old Javanese male, a farmer by occupation, was referred from a district hospital with complaints of lower abdominal discomfort, intermittent bloody diarrhea, and subtle alterations in bowel habits persisting over several weeks. He also experienced nausea, vomiting, and reported unintentional weight loss. His medical history was significant for a diagnosis of poorly differentiated adenocarcinoma of the ascending colon at the age of 31, for which he underwent curative resection with negative margins (R0) and had pathologically confirmed nodal involvement; the TNM staging was T3N2M0. Adjuvant chemotherapy with the FOLFOX regimen was completed over 6 months without complications, and serial follow-up imaging showed no evidence of disease recurrence. The patient remained asymptomatic and functional in his daily activities for 11 years after treatment.

For the surveillance, the patient has already undergone routine colonoscopy each year until the fifth year of follow-up. The abdominal computed tomography (CT) scan reported no distant organ metastasis. A chest X-ray was performed to evaluate the evidence of pulmonal metastasis, and during the follow-up after 5 years, there was no evidence of pulmonal metastasis.

**Figure 1:**
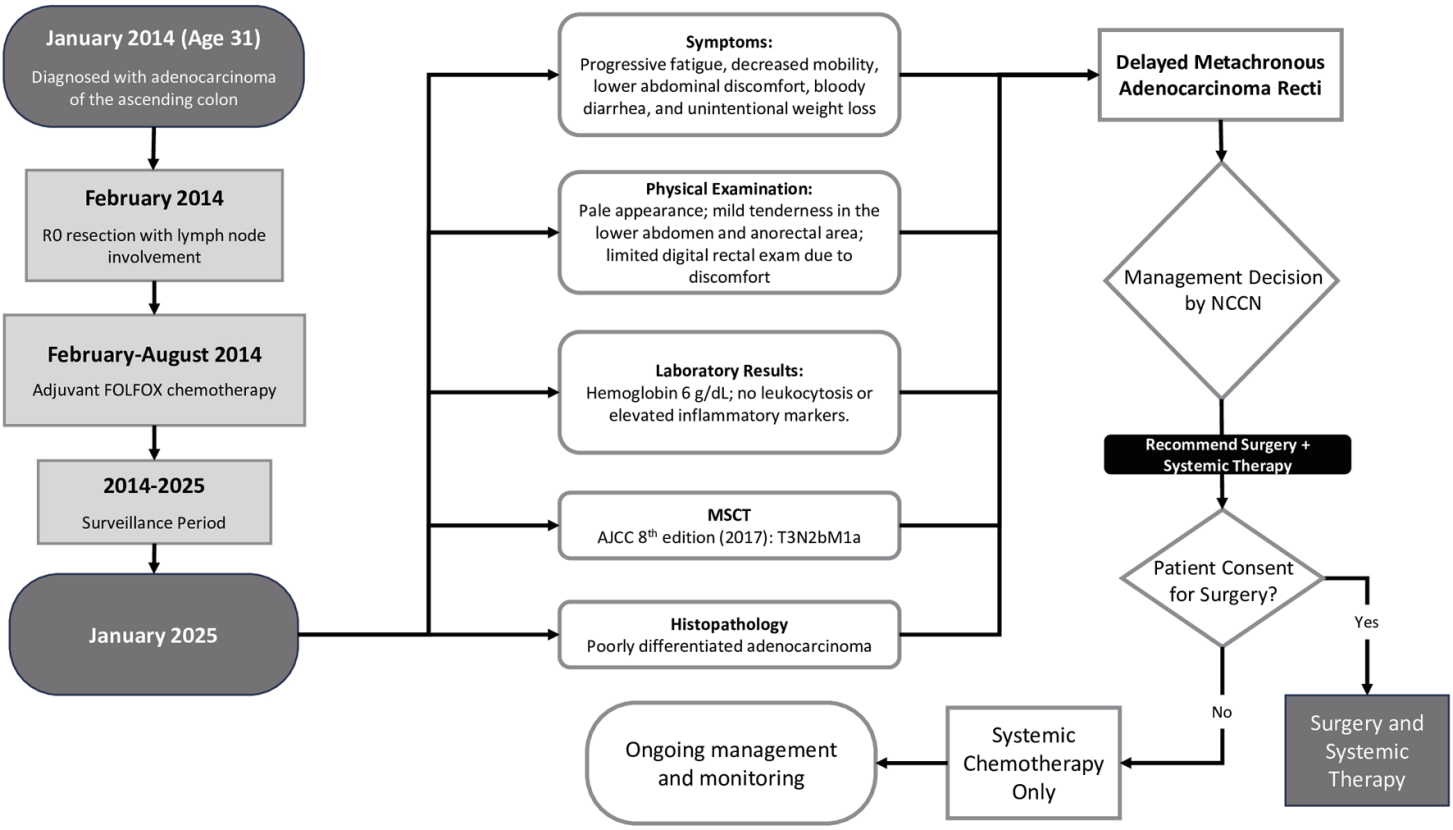
Timeline of patient.

In early 2025, the patient began to experience progressive fatigue and limited mobility. He denied any prior comorbidities and had no known family history of malignancy. Upon physical examination, he appeared pale with mild tenderness localized to the lower abdomen and anorectal area. Digital rectal examination was limited due to discomfort. Laboratory studies revealed marked anemia with a hemoglobin level of 6 g/dL but no leukocytosis or elevated inflammatory markers (Fig. 1) [6, 7].

**Figure 2:**
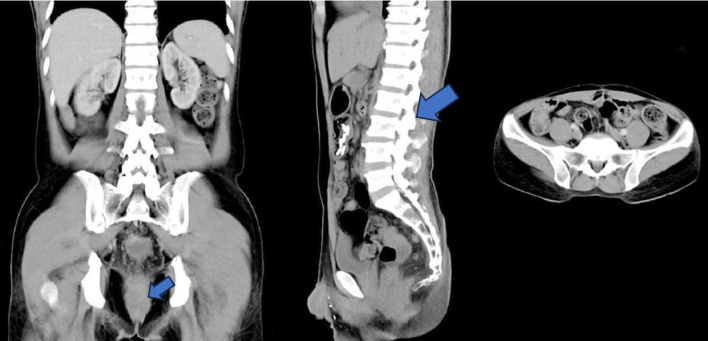
Multi-slice computed tomography image demonstrating a rectal mass extending from the level of the S2 vertebral body to the coccyx, infiltrating the submucosa, muscularis mucosa, and muscularis propria, with extension into the pericolorectal fat and associated fat stranding. Multiple enlarged lymph nodes are seen in the perivesical, pericolic, mesenteric, para-aortic, and bilateral inguinal regions. Subpleural pulmonary nodules are visible, suggestive of pulmonary metastases. Staging according to AJCC eighth edition (2017): T3N2bM1a (stage IVA) [7].

CT imaging of the abdomen and pelvis demonstrated a soft tissue mass in the rectal region, extending from the level of the second sacral vertebra to the coccyx, with suspected invasion into adjacent mesorectal tissue (Fig. 2). Clinical staging, using the AJCC eighth edition criteria, was determined as T3N2bM1a, indicating a potentially resectable stage IVa CRC.

An anoscopic evaluation was subsequently performed and biopsies from the rectal mass confirmed the diagnosis of moderately to poorly differentiated adenocarcinoma. The findings were consistent with a metachronous recurrence, appearing approximately 11 years after the initial curative surgery, well beyond the conventional 5-year surveillance window. This delayed recurrence is considered exceptionally rare in the context of EOCRC. Given the localized nature of the recurrence and absence of widespread metastatic disease, a multidisciplinary team recommended surgical resection followed by systemic therapy as per NCCN guidelines. However, the patient declined further surgery and consented only to systemic chemotherapy. He began first-line treatment with a FOLFOX regimen consisting of 5-fluorouracil, leucovorin, and oxaliplatin. At the time of reporting, the patient had completed four of the planned six cycles. Clinically, he showed partial response with decreased abdominal pain and fewer episodes of bloody stools. Treatment tolerance was favorable, with only mild nausea and fatigue reported, requiring no hospitalization or dose modification. The patient maintained independence in daily activities and continued to receive strong familial support throughout his treatment course.

This case study has already been approved by the Institutional Review Board of Health Research Ethic Committee of Moewardi General Hospital, Surakarta, Indonesia. Ethical clearance number is 132/IV/HREC/2025.

## Discussion

This case highlights a delayed metachronous recurrence of EOCRC 11 years after curative resection of a primary tumor in the ascending colon. Typically, metachronous recurrences are identified within 2 - 5 years following definitive treatment, making this case highly unusual and clinically significant. Such a presentation underlines the importance of long-term surveillance in EOCRC, which continues to be underestimated in current clinical practice.

The patient was initially managed appropriately with R0 surgical resection and 6 months of adjuvant FOLFOX chemotherapy, consistent with NCCN guidelines for stage III CRC [6]. He remained recurrence-free for 11 years, until presenting with a symptomatic rectal mass. Clinical staging indicated T3N2bM1a disease potentially resectable metastasis limited to one site which still fell under the category of potentially curable stage IVa CRC [7]. However, the patient declined further surgery, due to concerns about invasiveness, perceived surgical risk, or psychosocial barriers. As a result, management was limited to systemic chemotherapy, reducing the overall potential for curative intent.

Since 1994, EOCRC has shown a global annual increase of about 2% [1]. By 2030, it is estimated that 11% of colon cancers and 23% of rectal cancers will affect individuals younger than 50 years. Japan shows a contrasting trend, with a decrease in EOCRC incidence, possibly attributed to its early screening programs initiated at age 40 [2]. This subtype is recognized as biologically distinct from late-onset CRC, with stronger associations with genetic predisposition such as Lynch syndrome, MSI, and familial adenomatous polyposis [3]. Patients with EOCRC often present at more advanced stages and are more likely to exhibit poorly differentiated histopathology, including mucinous and signet ring subtypes [2]. These features support the aggressive behavior and unpredictable recurrence patterns seen in younger patients.

Cases with delayed metachronous recurrence in EOCRC are very rare. Most CRC recurrences occur in the first 2 - 3 years after curative surgery, and > 90% of metachronal cases appear within 5 years [4]. However, cases of relapse after > 5 years can still occur and are referred to as delayed metachronous recurrence. In a review by Broadbridge et al (2013), patients with CRC who relapsed after 5 years had no better clinical characteristics and survival than patients who relapsed at 2 - 5 years. This study is not limited to EOCRC, but provides an important framework that late relapse should not be overlooked [4]. Yagi et al (2023) reported two cases of adult colon cancer (62 and 52 years) with metachronous liver residicity after 9 and 15 years of recurrence-free [8]. Although patients were not strictly included in EOCRC, these cases showed the biological possibility of delayed recurrence even after the first decade postoperatively. To date, there have not been many specific reports of delayed metachronous recurrence in the EOCRC group. The available literature generally includes patients of all ages or only reports early recurrence. Therefore, cases of metachronal relapse 10 years after surgery in EOCRC patients such as those in this report are extremely rare and emphasize the importance of long-term surveillance and unusual tumor biological considerations. Within the limits of this research, this is the first case report to specifically report such a late recurrence particularly in young CRC population.

This case reinforces the limitations of fixed duration surveillance protocols and advocates for an individualized, risk-based follow-up strategy, particularly in younger patients with biologically aggressive tumors. The patient’s clinical course also underscores the importance of integrating molecular profiling, such as RAS/BRAF mutational analysis and MSI testing, in guiding personalized treatment and optimizing long-term outcomes [6].

The existing investigations on the molecular profile in EOCRC have yielded inconsistent findings. Current publications provide data that EOCRC tumors occur more frequently with MSI H. In these group, MSI tumors are predominantly connected with Lynch syndrome and infrequently with MLH1 inactivation, which is more common in older individuals, but are more often linked to MSH2 inactivation. They are linked to a more favorable outcome compared to microsatellite stable (MSS) tumors [9].

The MMR status possesses possible therapeutic implications. CRCs demonstrating MMR deficiency generally display a suboptimal response to fluorouracil-based adjuvant treatment. Notably, patients with MMR-deficient tumors generally demonstrate improved overall survival, which is ascribed to the tumors’ heightened immunogenicity [10].

Furthermore, this report emphasizes the critical role of shared decision making and patient education. The patient’s refusal of surgery in this case may have impacted his curative potential. Clear communication regarding risks, benefits, and alternatives must be prioritized to ensure that patients are empowered to make informed treatment choices.

In summary, delayed metachronous recurrence in EOCRC, though rare, remains a real possibility. Clinicians should remain vigilant even after extended disease-free intervals. Long-term follow-up, precision-based management, and patient-centered care are essential components in addressing the unique challenges posed by EOCRC.

### Conclusion

This case illustrates a rare and delayed metachronous recurrence of EOCRC, occurring 11 years after initial curative treatment. It underscores the importance of long-term, individualized surveillance beyond standard follow-up protocols, especially in younger patients with biologically aggressive disease. The patient’s refusal of potentially curative surgery in favor of systemic chemotherapy highlights the critical need for effective patient education and shared decision making. Clinicians must recognize that EOCRC carries a distinct risk profile, often associated with delayed and aggressive recurrence patterns. Future efforts should focus on enhancing surveillance strategies, integrating molecular profiling, and adopting personalized treatment approaches to improve outcomes in this growing patient population.

## Data Availability

The authors declare that data supporting the findings of this study are available within the article.

## References

[1] O'Reilly M, Linehan A, Krstic A, Kolch W, Sheahan K, Winter DC, Mc Dermott R (2023). Oncotherapeutic Strategies in Early Onset Colorectal Cancer. Cancers (Basel).

[2] Takada K, Hotta K, Kishida Y, Ito S, Imai K, Ono H (2023). Comprehensive Analysis of Early-onset Colorectal Cancer: A Review. J Anus Rectum Colon.

[3] Pellino G, Fuschillo G, Gonzalez-Sarmiento R, Marti-Gallostra M, Selvaggi F, Espin-Basany E, Perea J (2024). Risk of metachronous neoplasia in early-onset colorectal cancer: meta-analysis. BJS Open.

[4] Broadbridge VT, Karapetis CS, Beeke C, Woodman RJ, Padbury R, Maddern G, Kim SW (2013). Do metastatic colorectal cancer patients who present with late relapse after curative surgery have a better survival?. Br J Cancer.

[5] Godhi S, Godhi A, Bhat R, Saluja S (2017). Colorectal Cancer: Postoperative Follow-up and Surveillance. Indian J Surg.

[6] https://pubmed.ncbi.nlm.nih.gov/29262132/.

[7] Amin MB, Greene FL, Edge SB, Compton CC, Gershenwald JE, Brookland RK, Meyer L (2017). The Eighth Edition AJCC Cancer Staging Manual: Continuing to build a bridge from a population-based to a more "personalized" approach to cancer staging. CA Cancer J Clin.

[8] Yagi S, Takahashi M, Tsuji T, Yanagibashi S, Higashihara T, Ohtsuka H, Hayashi T (2023). Two cases of colorectal liver metastasis with residual liver recurrence after a long recurrence-free survival period. Surg Case Rep.

[9] Saraiva MR, Rosa I, Claro I (2023). Early-onset colorectal cancer: A review of current knowledge. World J Gastroenterol.

[10] REACCT Collaborative, Zaborowski AM, Abdile A, Adamina M, Aigner F, d'Allens L, Allmer C (2021). Characteristics of Early-Onset vs Late-Onset Colorectal Cancer: A Review. JAMA Surg.

